# Molecular Pathogenesis of Radiation-Induced Cell Toxicity in Stem Cells

**DOI:** 10.3390/ijms18122749

**Published:** 2017-12-18

**Authors:** Wonhee Hur, Seung Kew Yoon

**Affiliations:** 1 The Catholic University Liver Research Center & WHO Collaborating Center of Viral Hepatitis, College of Medicine, The Catholic University of Korea, Seoul 06591, Korea; wendyhur@catholic.ac.kr; 2 Department of Internal Medicine, Seoul St. Mary’s Hospital, College of Medicine, The Catholic University of Korea, Seoul 06591, Korea

**Keywords:** radiation therapy, radiation-induced toxicity, stem cell, cancer stem cell, resistant

## Abstract

Radiation therapy is an effective cancer therapy, but damage to normal tissues surrounding the tumor due to radiotherapy causes severe complications. The importance of the therapeutic area between tumor suppression and normal tissue injury has long been highlighted in radiation therapy. Recent advances in stem cell biology have shown that stem cell (SC) responses to genotoxic stresses of ionizing radiation can improve the therapeutic effect of radiation by repairing damaged cells. In contrast, cancer stem cells (CSCs), a small subpopulation of cells within tumors, are generally resistant to chemotherapy and radiotherapy and cause tumor recurrence. Although the underlying mechanisms are not clearly understood in detail, efforts are still underway to identify SC treatment or CSC resistant pathogenesis of DNA damage agents such as radiation therapy. In response to radiation, CSCs differ from normal SCs in their biological properties due to severe deregulation of the self-renewal ability in CSCs. Differences of cleavage mode, cell cycle characteristics, replication potential, and activation/inactivation of DNA damage treatment and cancer-specific molecular pathways between normal SCs and CSCs confer a malignant phenotype upon CSCs. However, further studies are needed to identify normal SC and CSC-specific targets. In this review, we summarize the current advances in research regarding how normal SCs and CSCs respond to ionizing radiation, with a special emphasis on cell toxicity, radiosensitivity, signaling networks, DNA damage response (DDR) and DNA repair. In addition, we discuss strategies to develop new diagnostic and therapeutic techniques for predicting responses to cancer treatment and overcoming radiation-related toxicity.

## 1. Introduction

Radiation therapy is a highly effective tool in cancer treatment widely used for a variety of malignant tumors [1,2,3]. Approximately 50% of all cancer patients undergo radiation treatment, resulting in a cure rate of about 40% [4]. At the end of the 19th century, numerous groundbreaking discoveries laid the foundation for medical radiation. Radiation therapy was initiated in 1895 with the discovery of X-rays by the physicist Wilhelm Conrad Roentgen [5]. Since then, the application of radiation has paralleled advancements in medicine, biology, physics, astronomy, materials science and engineering [6]. Radiation therapy today is one of the most common ways to treat cancer, combined with surgery and chemotherapy.

Radiation is used to destroy tumor cells with high physical energy sufficient to overcome the electron binding energy at an atom or a molecule. The ionizing radiation used for cancer treatment is largely based on the rationale that rapidly proliferating cancer cells are more sensitive to DNA damage responses than normal cells. The greatest challenge for radiation therapy is to increase the highest probability of cure with the least morbidity. To this end, the understanding of radiation dose and target volume over the years has been intensive and has consequently contributed to the development of radiation oncology. Recent advances in our understanding of radiation effects has been expanded widely as results of radiation-induced tumor cell death and various signaling pathways involved in sensitivity, resistance and further molecular sensors that can alter the tumor response to radiation.

Normal cells neighboring the tumor inevitably receive a considerable dose of ionizing radiation. This exposure can cause damage to healthy tissues, which may appear immediately or later, if the patient survives. Although radiation therapy prolongs patient survival, normal tissue damage caused by radiation remains an important clinical concern. It is therefore important to address or prevent cell cytotoxicity from normal tissue damage because the severity of radiation-induced toxicity can be worse than the initial lesions treated. The pathogenesis of normal tissue response to radiation is complex and involves different mechanisms such as DNA damage repair, cell death, inflammation, angiogenesis and matrix remodeling, depending on the radiation dose and time course [7].

Stem cells (SCs) generate the progeny of differentiated cells that give rise to the maintenance and growth of normal tissues. In recent years, SCs have shown significant implications for the progress in many fields of biotechnology, including cell-based regenerative therapies, drug testing and screening, disease modeling and side effects in radiotherapy. Among these, there is a growing interest in the biological response of stem cells to radiation treatment. It is also essential that SCs in damaged tissue have specific and effective DNA-damage response mechanisms to avoid the propagation of genetic lesions to all their progeny. In contrast, cancer stem cells (CSCs) play a critical role in the relapse and resistance to ionizing radiation that occur in various tumors. The features that CSCs share with SC are not only self-renewal and differentiation but also high tumorigenic potential, and CSCs are also called tumor-initiating cells. To understand the mechanism of radiation-induced toxicity, observation of molecular changes in SCs and CSCs due to radiation treatment will have an important influence on the development of clinical therapy.

This review will introduce the biological effects of radiation therapy and describe the pathogenic mechanism of radiation-induced toxicity in SCs and CSCs. In addition, promising target proteins for radiation sensitization in SCs and CSCs will also be highlighted.

## 2. Biologic Effects of Radiation Therapy

**Figure 1:**
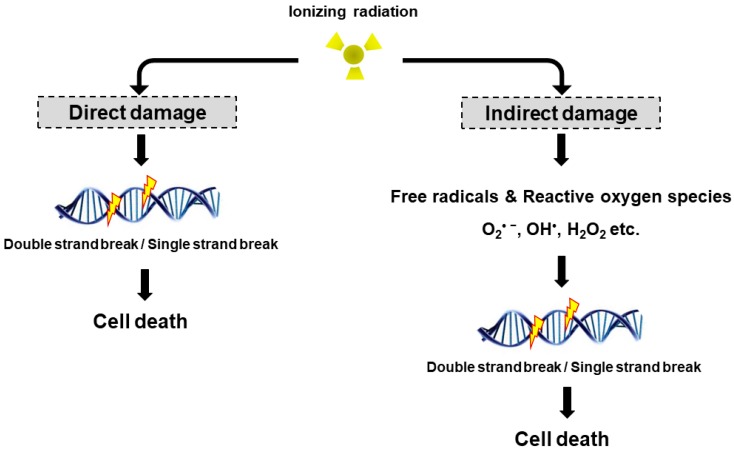
Direct and indirect DNA damage by ionizing radiation. Radiation can directly interact with cellular DNA and cause damage. The indirect DNA damage caused by the free radicals is derived from the ionization or excitation of the water component of the cells.

Radiation may cause DNA damage in the form of DNA strands breaks, either directly by ionizing the DNA or indirectly by forming free radicals that damage the DNA (Figure 1). DNA double strand breaks (DSBs) are biologically important lesions endogenously generated during replication or by reactive oxygen species (ROS). These DNA DSBs are repaired in higher eukaryotic cells by one of two distinct pathways, non-homologous end-joining (NHEJ) and homologous recombination (HR). HR repair is a key pathway to outcomes related to genome stability, during the late S phase to G2 phase of the mammalian cell cycle, as it leads to precise repair of DNA damage using the undamaged sister chromatid [8]. On the other hand, NHEJ repair is often imprecise because the DNA ends are modified before joining, leading to deletions or insertions at the break site [9]. DNA single strand breaks (SSBs) rarely cause cell death in normal cells since they are easily repaired by the repair system of the cell. Once damaged normal cells are properly repaired, the cells can survive without side effects due to DNA DSB. However, insufficient or incorrect DNA DSB repair can still lead to cell survival, indicating a genomic variation that can contribute to tumor formation. To maintain genomic integrity, cells detect damage signals, deliver to effector proteins and activate cell cycle arrest, the DNA repair pathway and cell death.

The biological effects of radiation treatment depend on the total dose, fractionation rate, radiosensitivity and linear energy transfer (LET) in tumors [10]. LET radiation destroys cancer by depositing the physical energy of radiation on the cancer cells, with the ionization density of its tracks quantified by the mean energy deposited per track length [11]. High LET radiation (neutrons and α-particles) results in stable free radicals and causes cellular damage from the direct ionization of cellular macromolecules, including DNA, RNA, lipids and proteins. High LET radiation induces complex DNA damage, a unique class of DNA lesions that include two or more individual lesions within the helical turns of the DNA molecule. These events are termed the “direct effect” (Figure 1). Conversely, low LET radiation (X-rays, γ-rays and β particles) generate free radicals and ROS, such as superoxide (O_2_^•−^), hydroxyl radical (OH^•^), as well as nonradical molecules such as hydrogen peroxide (H_2_O_2_) and singlet oxygen (^1^O_2_). Intracellular ROS can have many effects, including the oxidation of biological macromolecules and activation of intracellular signaling pathways. This effect enhances apoptosis and cell cycle arrest, subsequently leading to DNA DSBs or DNA SSBs in tumor cell [12], which is termed the “indirect effect” (Figure 1). The indirect effects also include phenomena such as the “bystander effect” [13]. Irradiated cells have been shown to release factors affecting adjacent cells that are not exposed to radiation, resulting in genomic instability, stress response and altered apoptosis or cell proliferation. Recently, Peng et al. identified a cathepsin B homolog CPR-4 as the major radiation-induced bystander effector inducing apoptosis inhibition and increased cell proliferation, lethality and stress response using the *Caenorhabditis elegans* (*C. elegans*) animal model [14]. In addition, the in vitro bystander effect is defined as a signal process that initiates from the irradiated cells and is transmitted to non-irradiated cells through “gap junction communication” [15,16,17] or “stress signaling factor (SSF)” released into the cell growth medium [18,19].

Based on studies on the biologic effects of radiation therapy, the technical improvement of radiotherapy over the years has been aimed at reducing the normal tissue impact and increasing tumor targets. Because direct DNA damage and indirect DNA damage caused by radiation are mechanically different from each other, a variety of new radiation sensitizers and protectants should be developed to correct for the two types of radiation reactions. To this end, it is important to study the mechanism of the radiation response and develop targeted drugs because the DNA damage response differs in different types of cells, particularly the stem cells of normal tissues and cancer stem cells of cancer tissues.

## 3. Mechanism of Radiation-Induced Cell Toxicity and Radiation Sensitization

Direct or indirect damage to DNA in the form of DNA breakage or replication stress collectively leads to a complex signaling system called the DNA damage response (DDR). DDRs include events that coordinate DNA repair, regulation of DNA replication, cell-cycle checkpoints, chromatin remodeling, associated regulation of various histone modifications and apoptosis [20]. Genome integrity in normal cells is ensured by efficient DDR signaling networks, including cell cycle checkpoints and DNA repair pathways. However, cancer cells may result from genomic instability and the accumulation of numerous genetic alterations. Therefore, to identify strategies to kill cancer cells with DNA-damaging agents without increasing normal cell toxicity, we must explore the differential response to DNA repair signaling between normal and tumor cells [21].

**Figure 2:**
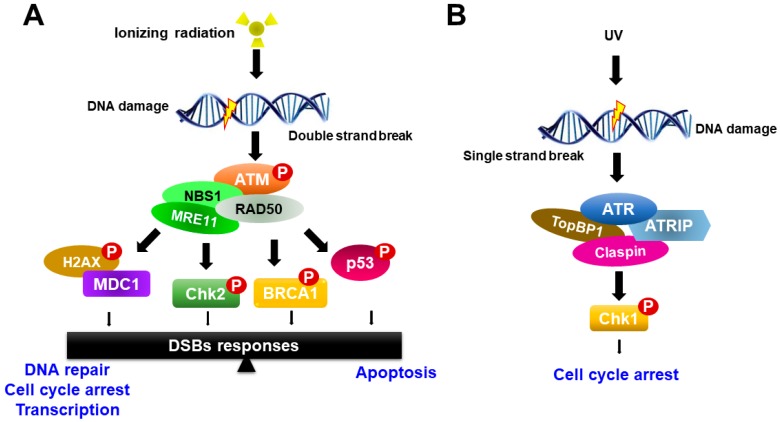
Schematic model for ATM and ATR activation in response to DNA damage. (**A**) ATM responds to DNA double-strand breaks and phosphorylates histone variant H2AX and nijmegen breakage syndrome 1 (NBS1), which localize to sites of DNA damage, where MRN complexes then form. ATM activation regulates cell-cycle checkpoints through the phosphorylation of Chk2, breast cancer type 1 (BRCA1) and p53, in addition to a wide number of other DDR factors, and the induction of the γH2AX-dependent signaling cascade. (**B**) ATR is activated in response to single-stranded DNA (ssDNA) by UV light. Activation of ATR requires DNA topoisomerase 2-binding protein 1 (TopBP1). ATR is recruited to replication protein A (RPA)-coated single-stranded DNA by its binding partner ATR Interacting Protein (ATRIP). ATR regulates the cell-cycle through activation of Chk1.

Radiation therapy induces chromosomal DNA lesions, resulting in the activation of the ataxia telangiectasia-mutated (ATM) and ATM-Rad3-related (ATR) protein kinases, which respond to DSBs and replication stress, respectively. The DDR network consists of two major parallel pathways that are controlled by the activation of ATM-serine-threonine checkpoint kinases 2 (Chk2) and ATR-Chk1 pathways (Figure 2). ATM and ATR large kinases trigger DNA damage response cascades, which phosphorylate and activate a variety of molecules to execute the DNA damage response and serve as key sensors for the entire DDR [22,23]. ATM and ATR share sequence similarity to lipid kinases of the phosphatidylinositol-3-kinase (PI3K) family but phosphorylate only protein substrates [20]. The DDR pathway is mediated by ATM and ATR as well as by two checkpoint effector kinases, Chk1 and Chk2, which are selectively phosphorylated and activated by ATM and ATR, respectively, to trigger a wide range of distinct downstream responses [23].

In response to ionizing radiation, ATM is recruited to the site of DNA damage and acts as a sensor that initiates ATM activation in conjunction with the MRE11-RAD50-NBS1 proteins (MRN complex). Activated ATM organizes repair of DSBs by phosphorylating numerous downstream targets, such as Chk2, H2AX, p53, mediator of DNA damage checkpoint protein 1 (MDC1), BRCA1 and the endonuclease CtIP [22,24]. Among phosphorylated targets, BRCA1 is involved in many cellular pathways that maintain genomic stability, including DNA double-strand break repair and DNA damage-induced cell cycle checkpoint activation. By contrast, the ATR-Chk1 pathway is activated most strongly upon DNA replication arrest, such as in the case of nucleotide depletion or replication-blocking DNA damage lesions caused by ultraviolet (UV) light. ATR is activated in response to single-stranded DNA (ssDNA) during DNA damage processing. ssDNA is recognized and coated with the trimeric ssDNA-binding protein complex Replication Protein A (RPA), which subsequently recruits and activates the ATR-ATRIP complex [25,26] (Figure 2). Other mediators of ATR activation in addition to ATRIP are the 9-1-1 complex, TopBP1 and Claspin. Subsequently, the activation of ATR induces Chk1 phosphorylation. Recent studies have shown that the activity of ATR-Chk1 is particularly important for the survival of cancer cells, which typically exhibit high levels of replication stress [27,28].

There is extensive crosstalk between the ATM-Chk2 and ATR-Chk1 pathways, which is affected by diverse factors, including types and doses of stress and timing. Many recent studies have shown that targeting of ATM or ATR might be a major concern in the development of therapeutic agents capable of controlling DNA damage without harming normal tissues. Agnihotri et al. identified a novel sensitizer to alkylating agents, 3-methylpurine-DNA glycosylase (MPG), using an siRNA screen targeting DNA damage response genes [29]. MPG, as a direct novel substrate of ATM, is phosphorylated and activated by ATM. They showed that the inhibition of MPG and ATM resulted in increased cytotoxic effects of alkylating agents in vitro and prolonged survival in vivo with no toxicity to normal cells, providing an ideal therapeutic window. In addition, ATM protein is a major player in signaling pathways classically implicated in stem cell maintenance as well as DNA damage processes. A recent study reported that ATM positively modulated the activity of ITCH E3-ubiquitin ligase [30]. ITCH is a member of the NEDD4-like family of HECT-E3-ubiquitin ligases, a family of proteins that participates in several physiological signaling pathways, including the DNA damage response, tumor necrosis factor (TNFα), Notch and Hedgehog signaling pathways. Fokas et al. demonstrated in vitro and in vivo radiosensitization of a highly selective ATR inhibitor (ATRi), VE-822 [31]. They showed that VE-822 decreased the survival of pancreatic cancer cells, but not normal cells, in response to radiation, suggested that ATRi can sensitize tumors to radiation without the cytotoxic effect of radiation on normal cells.

## 4. Response of Normal Stem Cells to Ionizing Radiation

**Figure 3:**
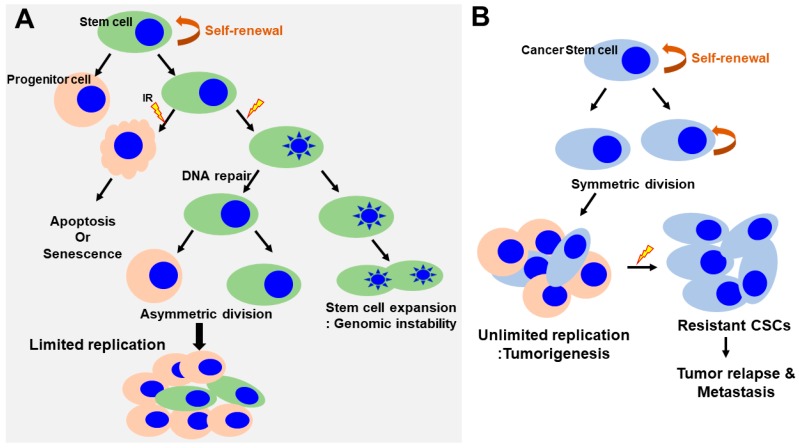
Regulation of self-renewal and DNA-damage response in normal and cancer stem cells. (**A**) Normal SCs asymmetrically divide, giving rise to stem cells and progenitor cells. Progenitor cells respond to radiation induced damage that induces apoptosis or senescence. SCs lead to the inactivation of apoptotic responses, cell cycle entry and expansion of the SC pool (increasing the rounds of symmetric divisions). Continuous DNA damage and repair suppress apoptosis/senescence, favoring the survival of SCs that harbor DNA mutations. This could have important pathological consequences by generating an actively expanding pool of immortal and genomically unstable SCs, increasing the risk of cancer. (**B**) In contrast, in cancer stem cells, the self-renewal capability is profoundly deregulated. The CSCs undergo an indefinite number of rounds of cell division, which, ultimately, results in the expansion of the stem cell pool. CSCs are a small but radioresistant cell subpopulation within heterogeneous cancer masses. Under conditions of radiation-induced stress, CSCs survive following IR. However, the majority of non-stem cancer cells are killed via various mechanisms, such as induction of cell apoptosis or mitotic death. A small number of non-stem cancer cells undergo dedifferentiation and transform into CSCs via unknown mechanisms. The newly generated CSCs, together with the intrinsic CSCs, subsequently contribute to the relapse and metastasis of cancer. CSCs, cancer stem cells; IR, irradiation.

The presence of human SCs has been demonstrated at various development stages in normal mammalian tissues, where these cells play a key role in maintaining tissue homeostasis during adult life. SCs are defined as undifferentiated cells that can self-renew through mitotic cell division to replenish the stem cell pool and differentiate into other cell types essential for tissue function [32,33,34]. SCs are broadly classified as either adult or embryonic. There is a difference in response to genotoxic stress, such as ionizing radiation between adult and embryonic stem cells. Human adult stem cells are characterized by a very heterogeneous response to radiation. Unlike, the mechanism of apoptosis in irradiation (IR)-exposed human embryonic stem cells have the tendency to undergo programmed cell death in response to genotoxic stress. As these SCs self-renew throughout life, accumulation of genetic mutations can damage genome integrity and potentially cause cancer (Figure 3A) [35]. Thus, SCs can be a major target for genetic and epigenetic alteration leading to radiation-induced toxicity [36,37]. The sensitivity of SCs to a genotoxic stress varies greatly depending on their type and developmental stage.

A previous study reported evidence for the existence of SCs through grafting experiments in lethally irradiated mice receiving transplanted bone marrow [38]. They demonstrated that supportive cells of the hematopoietic microenvironment contribute not only to the repopulation capacity of SCs but also to the maintenance of their quiescent state. Furthermore, the populations of bone-marrow stem cells have been implicated in the repair of ionizing irradiation damage of distant epithelial, as well as other hematopoietic, sites through their capacity to migrate through the circulation. This result suggests that the recovery of tissues and organs from radiation-induced toxicity is critically dependent on the repopulation of resident stem cells.

In addition, Jacobs et al. determined the relative radiosensitivity of undifferentiated normal SC populations compared with differentiated (non-stem) cells within several radiosensitive tissue niches and culture models [39]. They showed that the normal SCs were highly radiosensitive, whereas non-SCs were highly radioresistant and only underwent minimal apoptosis even at high doses, indicating that undifferentiated normal SCs exhibited an attenuated DDR and muted DNA repair (Figure 3A). However, these findings might not be applicable to all SC populations across every tissue niche, as the relative radioresistance of some SCs has been reported in other tissues [40,41]. They also demonstrated persistent phosphorylation of tyrosine 142 of histone H2AX (H2AX-Y142) and histone-3 lysine-56 acetylation (H3K56ac) at the DNA breaks in SCs despite abrogated ATM-mediated γH2AX induction, thereby inhibiting DDR signaling and promoting IR-induced apoptosis. These results suggested that the effects of H2AX-pY142 and H3K56ac on DDR signaling and IR-induced apoptosis provide evidence of pluralistic epigenetic regulation of stem cell radiosensitivity.

Similarly, the SC response to a genotoxic stress, such as ionizing radiation, must be a fine balance between maintenance of tissue homeostasis and genomic integrity. Based on many studies of the response of stem cell biology to ionizing irradiation, new therapeutic strategies, such as stem cell therapies for the treatment of radiation-induced normal tissue side effects, are current being developed.

## 5. Response of Cancer Stem Cells to Ionizing Radiation

Clinical and preclinical evidence suggests that radiation therapy may increase metastasis in both the primary tumor site and normal tissues under some circumstances [42,43]. For example, radiation therapy is one of the effective treatments for glioblastoma, but tumor renewal still leads to enhanced migratory and invasive behaviors of glioma cells, causing the eventual death of the patient. Conventional radiation therapy kills most cancer cells, but a subpopulation of cancer cells survive due to chemoresistance and radioresistance to therapy, eventually leading to tumor relapse and metastasis [44]. This subpopulation was termed cancer stem cells (CSCs) or tumor-initiating cells (TICs) within a tumor and possesses the capacity for self-renewing and generating a heterogeneous population of cancer cells composing the tumor (Figure 3B) [45,46,47]. CSCs can also reproduce to initiate tumors after xenotransplantation, thus enabling the maintenance of the entire tumor. Recent evidence suggests that CSCs may arise from normal stem cells, progenitor cells, or more differentiated cells through multiple mutations of genes due to their genomic instability or oncogene-induced plasticity. The genetic and epigenetic instability of these cells may result in the accumulation of mutations to gain self-renewal and tumorigenic abilities

The two main models of tumor development are the “stochastic clonal evolution” model and the “hierarchy (cancer stem cell)” model. According to the “stochastic clonal evolution” model, each cancer cells can be tumorigenic and maintain tumor growth [48]. However, in the “hierarchy (cancer stem cell)” model the tumor is organized hierarchically with a subpopulation of cells serving as CSCs, maintaining the tumor growth. The first CSC was identified in human acute myeloid leukemia (AML) by Dick and colleagues [49,50], who showed that a subpopulation of leukemia cells expressing a specific surface marker, CD34, but lacking the CD38 marker (CD34+/CD38−) was capable of repopulating the entire original disease over several transplantations, implying self-renewal and capacity to differentiate. Subsequently, the first solid CSCs were identified in breast tumors [46]. Al-Hajj et al. found that a subpopulation of breast cancer cells with a specific cell-surface antigen profile (CD44+/CD24−) can form tumors after subcutaneous injection into immunodeficient mice. In addition, the evidence for the existence of CSCs has been experimentally supported by findings from some hematological malignancies [51] and solid tumors [52,53,54,55,56,57,58,59,60]. To date, several surface markers for CSCs, including CD13 [61], CD24 [62], CD34 [63], CD38 [64], CD44 [65], CD 96 [66], CD133 [67], ALDH [68] and epithelial cell adhesion molecule (EpCAM) [69], have been reported. These markers enable CSCs to be distinguished from other tumor cells and normal stem cells.

The isolation and further characterization of CSCs from various human cancers are based on their immunogenicity and functional properties. Immunogenic CSCs were isolated using cell surface markers, while functional markers of CSCs are dependent on the surrogate characteristics of CSCs, including clonogenic growth, formation of tumor spheres, or resistance to chemo/radiotherapy (Figure 3B). Most studies on the isolation and functional properties of CSCs offer important and revolutionary advances in the targeting of cancer. Eradicating CSCs has been thought to be a promising approach to improving cancer survival rates. Thus, investigation of CSCs has been a hot spot of basic cancer research and are rapidly expanding into many related aspects of cancer research, including radiosensitization.

Radiation induces the CSC phenotype in many cancers, including breast, lung, liver and prostate cancers, as well as melanoma [70,71,72,73]. In liver cancer, ionizing irradiation confers CD133^+^ hepatocellular carcinoma (HCC) cells with a more aggressive tumor phenotype than CD133^−^ HCC cells, and the differentially expressed genes throughout the cellular processes are quite different between CD133^+^ and CD133^−^ cells after irradiation [72]. Additionally, increased cell proliferation in CD133^+^ HCC cells via the MAPK pathway by the upregulation of cell proliferation-related genes was also demonstrated. These results suggest that liver cancer cells expressing CD133 are associated with radioresistance and CSCs-targeting therapeutic approaches involving destruction of CD133^+^ liver CSCs may be promising for use in HCC treatment. Recent studies have suggested that IR increases the population of CD133^+^/CD44^+^ prostate CSCs and induces the re-expression of stem cell regulators, such as transcription factors Sox2, Oct4, Nanog and Klf4, which could be partially prevented by Notch inhibition [70]. Radiation-induced cell death occurs by a direct energy transfer to the DNA, mainly as a result of ROS generation. A previous study reported that radiotherapy results in low amounts of ROS in breast CSCs compared to non-CSCs. This decrease in the ROS level in breast tissue could be the result of increased radical scavenger properties. In addition, CSCs residing in the hypoxic regions, can be partly protected from radiation insults through decreased ROS generation and activation of the hypoxia-inducible factor-1 (HIF-1). HIF-1 is the master transcription factor, which is activated by hypoxic damage in tumor cells following radiotherapy promotes expression of genes that allow tumor cells to survive, leading to development of radioresistance [74,75]. Interestingly, heat-shock protein 90 (Hsp90), a highly conserved molecular chaperone, has been an attractive molecular target despite the lack of understanding of its role in CSCs. Recent study has shown that the HSP90 inhibitor, 17-*N*-allylamino-17-demethoxygeldanamycin, could effectively target thyroid CSC function in vitro and prevent migration and invasion [76]. These results suggest that HSP90 inhibitors may lead to important new therapeutic strategies to improve anticancer treatment and combat drug-resistance mechanisms in the future.

Recently, Kim et al. demonstrated that the increased clonogenic potential of liver CSCs is highly associated with deregulation of Wnt/β-catenin signaling and that Wnt/β-catenin signaling activity is positively correlated with CD133+/ALDH+ liver CSCs [77]. They also showed that Wnt/β-catenin small-molecule inhibitor CWP232228 suppresses the population of CD133+/ALDH+ liver CSCs, thus ultimately diminishing the self-renewal capacity of CSCs and decreasing tumorigenicity in vitro and in vivo. These results suggest that CWP232228 acts as a candidate therapeutic agent for liver cancer by preferentially targeting liver CSCs. Moreover, the Wnt/β-catenin small-molecule inhibitor acts as a candidate therapeutic agent not only for liver CSCs but also for different types of CSCs [78].

The Notch and Hedgehog signaling pathways play an essential role in embryogenesis and regulation of cellular processes, including proliferation, angiogenesis and self-renewal. Hong et al. recently reported that the subpopulation of CD133+ liver CSCs showed greater resistance to sublethal irradiation and enhanced cell invasion and migration capabilities [79]. They also showed that ADAM metallopeptidase domain 17 (ADAM17) silencing in ADAM17/Notch signaling significantly inhibited the migration and invasiveness of enriched CD133+ CSCs after irradiation. In conclusion, these findings indicate that suppression of ADAM17 shows promise for improving the efficiency of the current radiotherapies and reducing the metastatic potential of liver CSCs during HCC treatment. In addition, Ahmed et al. reported a variation in intra-patient genetic heterogeneity in liver metastases treated by radiation based on the formation and location of the primary tumor [80]. These results suggest that attempts to combine radiotherapy with cellular and molecular targeted biological modalities influences the sensitivity or resistance to ionizing radiation. These reports support that targeting CSCs or signaling pathway proteins may hold promise for developing novel combination modalities and overcoming radioresistance.

## 6. Conclusions

The advantages of radiation therapy include non-invasive treatment and organ preservation compared with surgery. However, the effect of radiation therapy is limited by the radioresistance of CSCs and by the radiation-induced damage to normal tissue cells located in the field of ionizing radiation. Radiation can also generate genome-destabilizing mutations that ultimately induce secondary cancers, leading to tumor recurrence and metastasis [81,82]. It is therefore important to develop new diagnostic and therapeutic techniques for predicting responses to cancer treatment and overcoming radiation-related toxicity. In addition, the use of stem cell therapy to promote the recovery of normal tissue exposed to ionizing radiation has important implications for studies to improve the unintended side effects of normal tissue injury. Successful transplantation of stem cells after high-dose chemotherapy or radiotherapy can lead to the production of new blood cells by the bone marrow. In some cases, newly produced blood cells may have the added benefit of attacking and destroying cancer cells that survived the initial treatment. However, recent studied have demonstrated that stem cell therapy has been proven to be associated with increased risk of second cancers from the chemotherapy and radiation used. Although certain pharmacological approaches are beneficial, the development of novel stem cell or cancer stem cell-based therapeutic approaches that can overcome radiation-induced toxicity remains a new but rapidly growing area of research.

## References

[1] Liauw S.L., Connell P.P., Weichselbaum R.R. (2013). New paradigms and future challenges in radiation oncology: An update of biological targets and technology. Sci. Transl. Med..

[2] Guadagnolo B.A., Liao K.P., Elting L., Giordano S., Buchholz T.A., Shih Y.C. (2013). Use of radiation therapy in the last 30 days of life among a large population-based cohort of elderly patients in the United States. J. Clin. Oncol..

[3] Perez C.A., Mutic S. (2013). Advances and future of Radiation Oncology. Rep. Pract. Oncol. Radiother..

[4] Baskar R., Lee K.A., Yeo R., Yeoh K.W. (2012). Cancer and radiation therapy: Current advances and future directions. Int. J. Med. Sci..

[5] Posner E. (1970). Reception of Rontgen’s discovery in Britain and U.S.A. Br. Med. J..

[6] Thariat J., Hannoun-Levi J.M., Sun Myint A., Vuong T., Gerard J.P. (2013). Past, present, and future of radiotherapy for the benefit of patients. Nat. Rev. Clin. Oncol..

[7] Bentzen S.M. (2006). Preventing or reducing late side effects of radiation therapy: Radiobiology meets molecular pathology. Nat. Rev. Cancer.

[8] Moynahan M.E., Jasin M. (2010). Mitotic homologous recombination maintains genomic stability and suppresses tumorigenesis. Nat. Rev. Mol. Cell Biol..

[9] Pellicioli A., Lee S.E., Lucca C., Foiani M., Haber J.E. (2001). Regulation of Saccharomyces Rad53 checkpoint kinase during adaptation from DNA damage-induced G2/M arrest. Mol. Cell.

[10] Hall E.J. (2007). Cancer caused by X-rays—A random event?. Lancet Oncol..

[11] Maier P., Hartmann L., Wenz F., Herskind C. (2016). Cellular Pathways in Response to Ionizing Radiation and Their Targetability for Tumor Radiosensitization. Int. J. Mol. Sci..

[12] Spitz D.R., Azzam E.I., Li J.J., Gius D. (2004). Metabolic oxidation/reduction reactions and cellular responses to ionizing radiation: A unifying concept in stress response biology. Cancer Metastasis Rev..

[13] Nagasawa H., Little J.B. (1992). Induction of sister chromatid exchanges by extremely low doses of alpha-particles. Cancer Res..

[14] Peng Y., Zhang M., Zheng L., Liang Q., Li H., Chen J.T., Guo H., Yoshina S., Chen Y.Z., Zhao X. (2017). Cysteine protease cathepsin B mediates radiation-induced bystander effects. Nature.

[15] Azzam E.I., de Toledo S.M., Little J.B. (2001). Direct evidence for the participation of gap junction-mediated intercellular communication in the transmission of damage signals from alpha -particle irradiated to nonirradiated cells. Proc. Natl. Acad. Sci. USA.

[16] Diegeler S., Hellweg C.E. (2017). Intercellular Communication of Tumor Cells and Immune Cells after Exposure to Different Ionizing Radiation Qualities. Front. Immunol..

[17] De Toledo S.M., Buonanno M., Harris A.L., Azzam E.I. (2017). Genomic instability induced in distant progeny of bystander cells depends on the connexins expressed in the irradiated cells. Int. J. Radiat. Biol..

[18] Le M., Fernandez-Palomo C., McNeill F.E., Seymour C.B., Rainbow A.J., Mothersill C.E. (2017). Exosomes are released by bystander cells exposed to radiation-induced biophoton signals: Reconciling the mechanisms mediating the bystander effect. PLoS ONE.

[19] Yin X., Tian W., Wang L., Wang J., Zhang S., Cao J., Yang H. (2015). Radiation quality-dependence of bystander effect in unirradiated fibroblasts is associated with TGF-beta1-Smad2 pathway and miR-21 in irradiated keratinocytes. Sci. Rep..

[20] Ciccia A., Elledge S.J. (2010). The DNA damage response: Making it safe to play with knives. Mol. Cell.

[21] Khanna A. (2015). DNA damage in cancer therapeutics: A boon or a curse?. Cancer Res..

[22] Waterworth W.M., Footitt S., Bray C.M., Finch-Savage W.E., West C.E. (2016). DNA damage checkpoint kinase ATM regulates germination and maintains genome stability in seeds. Proc. Natl. Acad. Sci. USA.

[23] Bartek J., Lukas J. (2003). Chk1 and Chk2 kinases in checkpoint control and cancer. Cancer Cell.

[24] Lee J.H., Paull T.T. (2005). ATM activation by DNA double-strand breaks through the Mre11-Rad50-Nbs1 complex. Science.

[25] Zou L., Elledge S.J. (2003). Sensing DNA damage through ATRIP recognition of RPA-ssDNA complexes. Science.

[26] Byun T.S., Pacek M., Yee M.C., Walter J.C., Cimprich K.A. (2005). Functional uncoupling of MCM helicase and DNA polymerase activities activates the ATR-dependent checkpoint. Genes Dev..

[27] Roos W.P., Thomas A.D., Kaina B. (2016). DNA damage and the balance between survival and death in cancer biology. Nat. Rev. Cancer.

[28] Jeggo P.A., Pearl L.H., Carr A.M. (2016). DNA repair, genome stability and cancer: A historical perspective. Nat. Rev. Cancer.

[29] Agnihotri S., Burrell K., Buczkowicz P., Remke M., Golbourn B., Chornenkyy Y., Gajadhar A., Fernandez N.A., Clarke I.D., Barszczyk M.S. (2014). ATM regulates 3-methylpurine-DNA glycosylase and promotes therapeutic resistance to alkylating agents. Cancer Discov..

[30] Stagni V., Santini S., Barila D. (2014). ITCH E3 ligase in ATM network. Oncoscience.

[31] Fokas E., Prevo R., Pollard J.R., Reaper P.M., Charlton P.A., Cornelissen B., Vallis K.A., Hammond E.M., Olcina M.M., Gillies McKenna W. (2012). Targeting ATR in vivo using the novel inhibitor VE-822 results in selective sensitization of pancreatic tumors to radiation. Cell Death Dis..

[32] Morrison S.J., Shah N.M., Anderson D.J. (1997). Regulatory mechanisms in stem cell biology. Cell.

[33] Ito K., Ito K. (2016). Metabolism and the Control of Cell Fate Decisions and Stem Cell Renewal. Annu. Rev. Cell Dev. Biol..

[34] Morrison S.J., Kimble J. (2006). Asymmetric and symmetric stem-cell divisions in development and cancer. Nature.

[35] Pardal R., Clarke M.F., Morrison S.J. (2003). Applying the principles of stem-cell biology to cancer. Nat. Rev. Cancer.

[36] Mohrin M., Bourke E., Alexander D., Warr M.R., Barry-Holson K., Le Beau M.M., Morrison C.G., Passegue E. (2010). Hematopoietic stem cell quiescence promotes error-prone DNA repair and mutagenesis. Cell Stem Cell.

[37] Beerman I. (2017). Accumulation of DNA damage in the aged hematopoietic stem cell compartment. Semin. Hematol..

[38] Greenberger J.S., Epperly M. (2009). Bone marrow-derived stem cells and radiation response. Semin. Radiat. Oncol..

[39] Jacobs K.M., Misri S., Meyer B., Raj S., Zobel C.L., Sleckman B.P., Hallahan D.E., Sharma G.G. (2016). Unique epigenetic influence of H2AX phosphorylation and H3K56 acetylation on normal stem cell radioresponses. Mol. Biol. Cell.

[40] Sugrue T., Brown J.A., Lowndes N.F., Ceredig R. (2013). Multiple facets of the DNA damage response contribute to the radioresistance of mouse mesenchymal stromal cell lines. Stem Cells.

[41] Solier S., Pommier Y. (2011). MDC1 cleavage by caspase-3: A novel mechanism for inactivating the DNA damage response during apoptosis. Cancer Res..

[42] Chang L., Graham P., Hao J., Ni J., Deng J., Bucci J., Malouf D., Gillatt D., Li Y. (2016). Cancer stem cells and signaling pathways in radioresistance. Oncotarget.

[43] Rycaj K., Tang D.G. (2014). Cancer stem cells and radioresistance. Int. J. Radiat. Biol..

[44] Kang M.K., Kang S.K. (2007). Tumorigenesis of chemotherapeutic drug-resistant cancer stem-like cells in brain glioma. Stem Cells Dev..

[45] Clarke M.F., Dick J.E., Dirks P.B., Eaves C.J., Jamieson C.H., Jones D.L., Visvader J., Weissman I.L., Wahl G.M. (2006). Cancer stem cells-perspectives on current status and future directions: AACR Workshop on cancer stem cells. Cancer Res..

[46] Al-Hajj M., Wicha M.S., Benito-Hernandez A., Morrison S.J., Clarke M.F. (2003). Prospective identification of tumorigenic breast cancer cells. Proc. Natl. Acad. Sci. USA.

[47] Bao S., Wu Q., McLendon R.E., Hao Y., Shi Q., Hjelmeland A.B., Dewhirst M.W., Bigner D.D., Rich J.N. (2006). Glioma stem cells promote radioresistance by preferential activation of the DNA damage response. Nature.

[48] Reya T., Morrison S.J., Clarke M.F., Weissman I.L. (2001). Stem cells, cancer, and cancer stem cells. Nature.

[49] Lapidot T., Sirard C., Vormoor J., Murdoch B., Hoang T., Caceres-Cortes J., Minden M., Paterson B., Caligiuri M.A., Dick J.E. (1994). A cell initiating human acute myeloid leukaemia after transplantation into SCID mice. Nature.

[50] Bonnet D., Dick J.E. (1997). Human acute myeloid leukemia is organized as a hierarchy that originates from a primitive hematopoietic cell. Nat. Med..

[51] Giambra V., Jenkins C.E., Lam S.H., Hoofd C., Belmonte M., Wang X., Gusscott S., Gracias D., Weng A.P. (2015). Leukemia stem cells in T-ALL require active Hif1alpha and Wnt signaling. Blood.

[52] Singh S.K., Hawkins C., Clarke I.D., Squire J.A., Bayani J., Hide T., Henkelman R.M., Cusimano M.D., Dirks P.B. (2004). Identification of human brain tumour initiating cells. Nature.

[53] Ricci-Vitiani L., Lombardi D.G., Pilozzi E., Biffoni M., Todaro M., Peschle C., De Maria R. (2007). Identification and expansion of human colon-cancer-initiating cells. Nature.

[54] Hermann P.C., Huber S.L., Herrler T., Aicher A., Ellwart J.W., Guba M., Bruns C.J., Heeschen C. (2007). Distinct populations of cancer stem cells determine tumor growth and metastatic activity in human pancreatic cancer. Cell Stem Cell.

[55] Eramo A., Lotti F., Sette G., Pilozzi E., Biffoni M., Di Virgilio A., Conticello C., Ruco L., Peschle C., De Maria R. (2008). Identification and expansion of the tumorigenic lung cancer stem cell population. Cell Death Differ..

[56] Collins A.T., Berry P.A., Hyde C., Stower M.J., Maitland N.J. (2005). Prospective identification of tumorigenic prostate cancer stem cells. Cancer Res..

[57] Yang Z.F., Ho D.W., Ng M.N., Lau C.K., Yu W.C., Ngai P., Chu P.W., Lam C.T., Poon R.T., Fan S.T. (2008). Significance of CD90+ cancer stem cells in human liver cancer. Cancer Cell.

[58] Schatton T., Murphy G.F., Frank N.Y., Yamaura K., Waaga-Gasser A.M., Gasser M., Zhan Q., Jordan S., Duncan L.M., Weishaupt C. (2008). Identification of cells initiating human melanomas. Nature.

[59] Curley M.D., Therrien V.A., Cummings C.L., Sergent P.A., Koulouris C.R., Friel A.M., Roberts D.J., Seiden M.V., Scadden D.T., Rueda B.R. (2009). CD133 expression defines a tumor initiating cell population in primary human ovarian cancer. Stem Cells.

[60] Li Y., Lin K., Yang Z., Han N., Quan X., Guo X., Li C. (2017). Bladder cancer stem cells: Clonal origin and therapeutic perspectives. Oncotarget.

[61] Haraguchi N., Ishii H., Mimori K., Tanaka F., Ohkuma M., Kim H.M., Akita H., Takiuchi D., Hatano H., Nagano H. (2010). CD13 is a therapeutic target in human liver cancer stem cells. J. Clin. Investig..

[62] Jinesh G.G., Manyam G.C., Mmeje C.O., Baggerly K.A., Kamat A.M. (2017). Surface PD-L1, E-cadherin, CD24, and VEGFR2 as markers of epithelial cancer stem cells associated with rapid tumorigenesis. Sci. Rep..

[63] Ishikawa F., Yoshida S., Saito Y., Hijikata A., Kitamura H., Tanaka S., Nakamura R., Tanaka T., Tomiyama H., Saito N. (2007). Chemotherapy-resistant human AML stem cells home to and engraft within the bone-marrow endosteal region. Nat. Biotechnol..

[64] Liu X., Grogan T.R., Hieronymus H., Hashimoto T., Mottahedeh J., Cheng D., Zhang L., Huang K., Stoyanova T., Park J.W. (2016). Low CD38 Identifies Progenitor-like Inflammation-Associated Luminal Cells that Can Initiate Human Prostate Cancer and Predict Poor Outcome. Cell Rep..

[65] Zhu Z., Hao X., Yan M., Yao M., Ge C., Gu J., Li J. (2010). Cancer stem/progenitor cells are highly enriched in CD133+CD44+ population in hepatocellular carcinoma. Int. J. Cancer.

[66] Hosen N., Park C.Y., Tatsumi N., Oji Y., Sugiyama H., Gramatzki M., Krensky A.M., Weissman I.L. (2007). CD96 is a leukemic stem cell-specific marker in human acute myeloid leukemia. Proc. Natl. Acad. Sci. USA.

[67] Ma S., Chan K.W., Hu L., Lee T.K., Wo J.Y., Ng I.O., Zheng B.J., Guan X.Y. (2007). Identification and characterization of tumorigenic liver cancer stem/progenitor cells. Gastroenterology.

[68] Ginestier C., Hur M.H., Charafe-Jauffret E., Monville F., Dutcher J., Brown M., Jacquemier J., Viens P., Kleer C.G., Liu S. (2007). ALDH1 is a marker of normal and malignant human mammary stem cells and a predictor of poor clinical outcome. Cell Stem Cell.

[69] Yamashita T., Ji J., Budhu A., Forgues M., Yang W., Wang H.Y., Jia H., Ye Q., Qin L.X., Wauthier E. (2009). EpCAM-positive hepatocellular carcinoma cells are tumor-initiating cells with stem/progenitor cell features. Gastroenterology.

[70] Lagadec C., Vlashi E., Della Donna L., Dekmezian C., Pajonk F. (2012). Radiation-induced reprogramming of breast cancer cells. Stem Cells.

[71] Koren A., Rijavec M., Kern I., Sodja E., Korosec P., Cufer T. (2016). BMI1, ALDH1A1, and CD133 Transcripts Connect Epithelial-Mesenchymal Transition to Cancer Stem Cells in Lung Carcinoma. Stem Cells Int..

[72] Piao L.S., Hur W., Kim T.K., Hong S.W., Kim S.W., Choi J.E., Sung P.S., Song M.J., Lee B.C., Hwang D. (2012). CD133+ liver cancer stem cells modulate radioresistance in human hepatocellular carcinoma. Cancer Lett..

[73] Rybak A.P., Bristow R.G., Kapoor A. (2015). Prostate cancer stem cells: Deciphering the origins and pathways involved in prostate tumorigenesis and aggression. Oncotarget.

[74] Peitzsch C., Perrin R., Hill R.P., Dubrovska A., Kurth I. (2014). Hypoxia as a biomarker for radioresistant cancer stem cells. Int. J. Radiat. Biol..

[75] Masoud G.N., Li W. (2015). HIF-1alpha pathway: Role, regulation and intervention for cancer therapy. Acta Pharm. Sin. B.

[76] White P.T., Subramanian C., Zhu Q., Zhang H., Zhao H., Gallagher R., Timmermann B.N., Blagg B.S., Cohen M.S. (2016). Novel HSP90 inhibitors effectively target functions of thyroid cancer stem cell preventing migration and invasion. Surgery.

[77] Kim J.Y., Lee H.Y., Park K.K., Choi Y.K., Nam J.S., Hong I.S. (2016). CWP232228 targets liver cancer stem cells through Wnt/beta-catenin signaling: A novel therapeutic approach for liver cancer treatment. Oncotarget.

[78] Jang G.B., Hong I.S., Kim R.J., Lee S.Y., Park S.J., Lee E.S., Park J.H., Yun C.H., Chung J.U., Lee K.J. (2015). Wnt/beta-Catenin Small-Molecule Inhibitor CWP232228 Preferentially Inhibits the Growth of Breast Cancer Stem-like Cells. Cancer Res..

[79] Hong S.W., Hur W., Choi J.E., Kim J.H., Hwang D., Yoon S.K. (2016). Role of ADAM17 in invasion and migration of CD133-expressing liver cancer stem cells after irradiation. Oncotarget.

[80] Ahmed K.A., Caudell J.J., El-Haddad G., Berglund A.E., Welsh E.A., Yue B., Hoffe S.E., Naghavi A.O., Abuodeh Y.A., Frakes J.M. (2016). Radiosensitivity Differences Between Liver Metastases Based on Primary Histology Suggest Implications for Clinical Outcomes After Stereotactic Body Radiation Therapy. Int. J. Radiat. Oncol. Biol. Phys..

[81] Sigurdson A.J., Jones I.M. (2003). Second cancers after radiotherapy: Any evidence for radiation-induced genomic instability?. Oncogene.

[82] Hegemann N.S., Schlesinger-Raab A., Ganswindt U., Horl C., Combs S.E., Holzel D., Gschwend J.E., Stief C., Belka C., Engel J. (2017). Risk of second cancer following radiotherapy for prostate cancer: A population-based analysis. Radiat. Oncol..

